# *Legionella *spp. and legionellosis in southeastern Italy: disease epidemiology and environmental surveillance in community and health care facilities

**DOI:** 10.1186/1471-2458-10-660

**Published:** 2010-11-02

**Authors:** Christian Napoli, Fabrizio Fasano, Roberta Iatta, Giovanna Barbuti, Teresa Cuna, Maria Teresa Montagna

**Affiliations:** 1 Department of Biomedical Science and Human Oncology - Hygiene section, University of Bari, Piazza G. Cesare 11, 70124 Bari, Italy; 2 Legionella Reference Laboratory - Regional Centre for Epidemiology (OER), Apulia Italy, Piazza G. Cesare 11, 70124 Bari, Italy

## Abstract

**Background:**

Following the publication of the Italian Guidelines for the control and prevention of legionellosis an environmental and clinical surveillance has been carried out in Southeastern Italy. The aim of the study is to identify the risk factors for the disease, so allowing better programming of the necessary prevention measures.

**Methods:**

During the period January 2000 - December 2009 the environmental surveillance was carried out by water sampling of 129 health care facilities (73 public and 56 private hospitals) and 533 buildings within the community (63 private apartments, 305 hotels, 19 offices, 4 churches, 116 gyms, 3 swimming pools and 23 schools). Water sampling and microbiological analysis were carried out following the Italian Guidelines. From January 2005, all facilities were subject to risk analysis through the use of a standardized report; the results were classified as *good *(G), *medium *(M) and *bad *(B). As well, all the clinical surveillance forms for legionellosis, which must be compiled by physicians and sent to the Regional Centre for Epidemiology (OER), were analyzed.

**Results:**

*Legionella *spp. was found in 102 (79.1%) health care facilities and in 238 (44.7%) community buildings. The percentages for the contamination levels < 1,000, 1,000-10,000, > 10,000 cfu/L were respectively 33.1%, 53.4% and 13.5% for samples from health care facilities and 33.5%, 43.3% and 23.2% for samples from the community. Both in hospital and community environments, *Legionella pneumophila *serogroup (*L. pn *sg) 2-14 was the most frequently isolate (respectively 54.8% and 40.8% of positive samples), followed by *L. pn *sg 1 (respectively 31.3% and 33%). The study showed a significant association between M or B score at the risk analysis and *Legionella *spp. positive microbiological test results (p < 0.001). From clinical surveillance, during the period January 2001 - August 2009, 97 cases of legionellosis were reported to the OER: 88 of community origin and 9 nosocomial. The most frequent symptoms were: fever (93.8%), cough (70.1%), dyspnea (58.8%), shivering (56.7%). Radiological evidence of pneumonia was reported in 68%. The laboratory diagnostic methods used were: urinary antigen (54.3%), single antibody titer (19.8%), only seroconversion (11.1%), other diagnostic methods (14.8%).

**Conclusions:**

Our experience suggests that risk analysis and environmental microbiological surveillance should be carried out more frequently to control the environmental spread of *Legionella *spp. Furthermore, the laboratory diagnosis of legionellosis cannot be excluded only on the basis of a single negative test: some patients were positive to only one of the diagnostic tests.

## Background

*Legionella *spp. is a ubiquitous intracellular microorganism present in natural and artificial water systems, which grows at temperatures 25-42°C, especially if the water is stagnant [1,2].

Legionellosis normally occurs after inhaling an aerosol containing *Legionella *bacteria produced from contaminated water sources such as cooling towers, hot water systems, showers, whirlpool spas, and similar disseminators that draw upon a water supply. As there have been no reported cases of interhuman transmission, it can be presumed that the environment is the only source of the infection. Individual reactions and the level of a person's susceptibility to the same source of infection depend on individual factors or already present pathologies.

With regard to epidemiological surveillance, the *European Working Group for Legionella Infections *(EWGLI), with 29 member states, was formed in 1986 to carry out international surveillance of travel-associated Legionnaires' disease [3]. From 1993 to March 2010 the EWGLI was coordinated by the Health Protection Agency (London, England); in April 2010, the scheme was transferred to the European Centre for Disease Prevention and Control (ECDC). It is now named *European Legionnaires' Disease Surveillance Network *(ELDSNet), and it involves all EU Member States, Iceland and Norway.

In Italy, national surveillance of the disease was established in 1983 and reporting became compulsory in 1990. Since then, a steady increase in both sporadic and epidemic cases has been reported, but if this upsurge is real or due to better reporting or improved ascertainment and changes in clinical methods of diagnosis is difficult to distinguish [4,5].

In the year 2000, the Italian Institute of Health (ISS), produced the first Guidelines on the control and prevention of legionellosis [6]; followed, in 2005, by instructions for laboratories with a role in microbiological diagnosis and environmental control [7] and for tourist accommodation and spas [8]. The instructions recommend that the measures which influence the growth and diffusion of *Legionella *spp. must be taken into account in the design and maintenance of water systems. Although it cannot be guaranteed that the bacteria will be completely eradicated, such measures reduce possible contamination.

Southeastern Italy is made up in the greatest part by the Apulia Region, with a land surface of 19,347 sq. km, and its Regional Center for Epidemiology (OER) is responsible for epidemiological surveillance and diseases control. Following the publication of the Guidelines, the OER incorporated the surveillance of *Legionella *spp. into its programs. Since then, environmental and clinical surveillance has been carried out on the whole of this part of Italy and the information obtained has been maintained in a computer database with real-time availability of all the information relative to the distribution of *Legionella *spp. contamination, so allowing better programming of the necessary measures for prevention and control. At the same time, all the isolated environmental and clinical strains of *Legionella *spp. have been collected for further bio-molecular and philogenetic studies to determine the most common genotypes. Herein are the results of the Southeastern Italy surveillance program.

## Methods

### Environmental sampling

During the period January 2000 - December 2009 the environmental surveillance was carried out on water sampling coming from 129 health care facilities (73 public and 56 private hospital) and 533 buildings within the community (305 hotels, 116 gyms, 63 private apartments, 23 schools, 19 offices, 4 churches, 3 swimming pools). In total, 13,286 water samples were analyzed: 7,148 from health-care facilities and 6,138 from the community. Water samples collected during re-inspections or after taking corrective actions were not included in the analysis. The water sampling was executed by inspectors of the Regional Agency for the Environment (ARPA) and the Local Health Units (AUSLs) in the presence of a member of the OER staff.

Following the Italian Guidelines [6], in each building, water samples of 1 liter were obtained from:

-incoming cold water, for a total of 635 samples;

-hot water systems (hot water leaving the water heater; circulating hot water returning to the heater; the most distant sites within the distribution system), for a total of 1,628 samples;

-rooms on different floors to be representative of the different loops of the distribution system (at least 10% of the total number of rooms): one sample of hot water was taken from shower and one from basin taps immediately after they were switched on to be representative of the colonization of the outlet, for a total of 10,329 samples;

-cold water cisterns, for a total of 224 samples;

-cooling towers (inc. a sample from the cooling tower pond), for a total of 460 samples;

-swimming pools, for a total of 3 samples;

-decorative fountains, for a total of 7 samples.

Sterile containers containing sodium thiosulphate to neutralize any oxidising biocide were used.

### Microbiological examination

The samples collected were kept at ambient temperature and protected from direct light during transport to the *Legionella Reference Laboratory *(Quality certified according to standard ISO 9001:2008), where, in accordance with the methods indicated in the Italian Guideline, they were submitted to filtration using 0.2 μm isopore polycarbonate membranes (Millipore Corporation, Bedford, MA, USA); these were then resuspended in 10 ml of the same water sample and vortexed: 5 ml were treated at 50°C for 30 min and seeded (0.1 ml) on GVPC medium. The remaining 5 ml were cold seeded using the same technique. After incubation at 36°C for 8-10 days in a damp environment at 2.5% CO^2^, quantitative assessment was made, expressed in cfu/l. The suspect colonies were subcultured on CYE medium and BCYE medium and those ascribable to the *Legionella *genus were serologically identified. As recommended by the EWGLI and Italian Guidelines, the laboratory applied a minimum theoretical mathematical detection limit equal to 100 *Legionella *bacteria per liter of sample. Samples equal to or greater than this value (100 ufc/l) were considered positive; buildings with *Legionella *spp. count > 100 ufc/l in at least one sample were also considered positive.

From 2006, in health-care facilities, *Legionella *spp. isolates were identified and serotyped using monovalent antisera.

When possible, to identify the precise source of infection, Pulsed-field gel electrophoresis (PFGE) was used to compare patient and environmental isolates, using NotI low-cutting enzyme (Roche, Italy).

### Risk analysis

From January 2005, all community and health care facilities underwent risk analysis during water sampling. The risk assessment was carried out by a member of the OER staff (trained at the 1^st ^EWGLI training course "Investigating outbreaks of legionnaires' disease: risk assessment, sampling and control", November 2004, Health Protection Agency, London).

As recommended by the Italian and EWGLI Guidelines, after a full inspection of the enrolled buildings to identify and evaluate potential source of risk, a standardized short report was completed.

The report, developed by the OER staff, included 18 items. For each item a score was assigned ranging from 1 (*very good*) to 3 (*very bad*). A total score ranging from 18 to 29, with no *very bad *score reported, was classified as *good *(G); a total score ranging from 30 to 41 was classified as *medium *(M) and a total score of 42 or over was classified as *bad *(B). As previously described *by Hadjichristodoulou et al*. [9] association between inspection results and facilities testing positive for *Legionella *spp. from water supply systems (with at least one sample with bacteria count > 10,000 or more than 2 samples with bacteria count > 1,000 but < 10,000) was assessed by a correlation analysis: Relative Risk (RR) and 95% confidence interval (CI) were calculated using software epi-info 2000.

### Clinical Surveillance

A part from the compulsory reporting of infectious disease, the Italian Guideline [6] states that for each case of legionellosis, a surveillance form must be completed by physicians and sent to the OER. The form reports observational information such as: laboratory diagnosis criteria, the origin (either acquired in the community or in hospital), the patient's personal details, risk factors, period of symptoms onset, symptoms and patient lifestyle prior to the disease. In the present study all the reports for the period January 2001 - August 2009 were analyzed.

Case definition [6]: an acute lower respiratory infection with focal signs of pneumonia on clinical examination and/or radiological evidence of pneumonia and one or more of the following laboratory tests:

-the presence of *Legionella *spp. urinary antigen (Ag);

-seroconversion (a four-fold or greater increase in titer, after at least 20-30 days) or at least one antibody (Ab) titre > 1:512, when it is not possible to evaluate the seroconversion because the first serum specimen at the onset of symptoms was not available;

-isolation (culture) of *Legionella *spp. from clinical specimens.

In addition, the principal characteristics of nosocomial and community cases in Southeastern Italy were compared (year 2001-2009), as were also regional and national nosocomial cases (year 2001-2007). The data for the national cases were obtained from the annual reports on legionellosis issued by the ISS, that at the time of the analysis were available to 2007 [10-16].

Statistical analysis was executed by the program Analyse-it v.1.71 (free trial) and Student's t-test was used to compare paired data. The relative confidence intervals at 95% were calculated and a value of p < 0.05 was considered significant for all the tests. Since no experiments on Humans were done and we analyzed epidemiological observational data from the National surveillance system for *Legionella *spp. infections [6], no ethical approval was required.

## Results

### Environmental Surveillance

*Legionella *spp. was found in 33.6% of the samples and 58.2% of the buildings enrolled (Table 1). For each kind of building, the total percentage of samples testing positive for *Legionella *spp. were, from basin taps (33.4%), showers (36.4%), incoming cold water points (23%), hot water system points (34.8%), cold water cisterns (34.4%), cooling towers (15.9%), pools (0) and decorative fountains (28.6%) (Table 2). *Legionella *spp. count was < 1,000 (33.3%), 1,000-10,000 (48.8%), > 10,000 cfu/L (17.9%) (Table 3), and the identified species were: *Legionella pneumophila *serogroup (*L. pn *sg) 1 (32.1%); *L. pn *sg 2-14 (48.4%); *L. species (L. longbeachae, L. bozemanii, L. dumoffii, L. gormanii, L. jordanis, L. micdadei, L. anisa) *(5.2%); *mixed cultures *(14.3%) (Table 4).

**Table 1:** Number of facilities/samples enrolled and tested positive for *Legionella *spp. (%)

Facilities		*N. Enrolled facilities*	*N. samples*	*N. facilities positive for Legionella spp. (%)*	*N. samples positive for Legionella spp. (%)*
**Health care**	Public Hospitals	73	6361	68 (93.2)	2250 (35.4)
	
	Private Hospitals	56	787	34 (60.7)	176 (22.4)
	
	**Sub-total**	**129**	**7148**	**102 (79.1)**	**2426 (33.9)**

**Community**	Apartments	63	189	34 (54)	81 (42.9)
	
	Hotels	305	5009	204 (66.9)	1826 (36.5)
	
	Offices	19	270	10 (52.6)	78 (28.9)
	
	Churches	4	53	1 (25)	3 (5.7)
	
	Gyms	116	472	30 (25.9)	46 (9.7)
	
	Swimming Pools	3	17	1 (33.3)	1 (5.9)
	
	Schools	23	128	3 (13)	3 (2.3)
	
	**Sub-total**	**533**	**6138**	**283 (53.1)**	**2038 (33.2)**

**TOTAL**		**662**	**13.286**	**385 (58.2)**	**4464 (33.6)**

**Table 2:** Distribution of samples tested positive for *Legionella spp*. by watersides

Facilities		N. positive taps/N. sampled taps (%)	N. positive showers/N. sampled showers (%)	N. positive incoming cold water points/N. sampled incoming cold water points (%)	N. positive hot water systemic points/N. positive hot water systemic points (%)	N. positive cold water cisterns/N. sampled cold water cisterns (%)	N. positive cooling towers/N. sampled cooling towers (%.)	N. positive pool basins/N. sampled pool basins (%)	N. positive fountain basins/N. sampled fountain basins (%)	Total
**Health care**	Public Hospitals	1106/3074 (36)	972/2774 (35)	19/73 (26)	136/341 (39.9)	3/10 (30)	14/88 (15.9)	0	0/1 (0)	2250/6361 (35.4)
	
	Private Hospitals	61/258 (23.6)	52/207 (25.1)	11/60 (18.3)	42/191(22)	4/16 (25)	6/55 (10.9)	0	0	176/787 (22.4)
	
	**Sub-total**	**1167/3332 (35)**	**1024/2981 (34.4)**	**30/133 (22.6)**	**178/532 (33.5)**	**7/26 (26.9)**	**20/143 (14)**	**0**	**0/1 (0)**	**2426/7148 (33.9)**

**Community**	Apartments	22/63 (34.9)	34/62 (54.8)	16/41 (39)	0	9/23 (39.1)	0	0	0	81/189 (42.9)
	
	Hotels	587/1709 (34.3)	688/1649 (41.7)	92/306 (30.1)	358/928 (38.6)	49/127 (38.6)	51/285 (17.9)	0	1/5 (20)	1826/5009 (36.5)
	
	Offices	29/124 (23.4)	16/44 (36.4)	4/19 (21.1)	20/59 (33.9)	6/12 (50)	2/11 (18.2)	0	1/1 (100)	78/270 (28.9)
	
	Churches	3/31 (9.7)	0/2 (0)	0/4 (0)	0/14 (0)	0/2 (0)	0	0	0	3/53 (5.7)
	
	Gyms	7/126 (5.6)	18/129 (14)	4/106 (3.8)	11/68 (16.2)	6/22 (27.3)	0/21 (0)	0	0	46/472 (9.7)
	
	Swimming pools	0/3 (0)	1/5 (20)	0/3 (0)	0/3 (0)	0	0	0/3 (0)	0	1/17 (5.9)
	
	Schools	0/46 (0)	3/23 (13)	0/23 (0)	0/24 (0)	0/12 (0)	0	0	0	3/128 (2.3)
	
	**Sub-total**	**648/2102 (30.8)**	**760/1914 (39.7)**	**116/502 (23.1)**	**389/1096 (35.5)**	**70/198 (35.4)**	**53/317 (16.7)**	**0/3 (0)**	**2/6 (33.3)**	**2038/6138 (33.2)**

**TOTAL**		**1815/5434 (33.4)**	**1784/4895 (36.4)**	**146/635 (23)**	**567/1628 (34.8)**	**77/224 (34.4)**	**73/460 (15.9)**	**0/3 (0)**	**2/7 (28.6)**	**4464/13286 (33.6)**

**Table 3:** Distribution of samples tested positive for *Legionella spp*. by count level (100-1000; > 1000 -10000; > 10000 cfu/L)

Facilities		100-1000 cfu/L	> 1000 - 10000 cfu/L	> 10000 cfu/L
**Health - care**	Public Hospitals	733/2250 (32.6)	1217/2250 (54.1)	300/2250 (13.3)
	
	Private Hospitals	70/176 (39.8)	79/176 (44.9)	27/176 (15.3)
	
	**Sub-total**	**803/2426 (33.1)**	**1296/2426 (53.4)**	**327/2426 (13.5)**

**Community**	Apartments	43/81 (53.1)	27/81 (23.3)	11/81 (13.6)
	
	Hotels	559/1826 (30.6)	815/1826 (44.6)	452/1826 (24.8)
	
	Offices	46/78 (59.0)	27/78 (34.6)	5/78 (6.4)
	
	Churches	1/3 (33.3)	1/3 (33.3)	1/3 (33.3)
	
	Gyms	31/46 (67.4)	12/46 (26.1)	3/46 (6.5)
	
	Swimming pools	1/1 (100)	0/0 (0)	0/0 (0)
	
	Schools	2/3 (66.7)	1/3 (33.3)	0/0 (0)
	
	**Sub-total**	**683/2038 (33.5)**	**883/2038 (43.3)**	**472/2038 (23.2)**

**Total**		**1486/4464 (33.3)**	**2179/4464 (48.8)**	**799/4464 (17.9)**

**Table 4:** Distribution of samples tested positive by species (*L. pn*.1, *L. pn*.2-14, L. species*, mixed cultures)

Facilities		N. samples positive *for L. pn.1* /N. samples positive for L. spp (%)	N. samples positive *for *L. *pn.2-14* /N. samples positive for L. spp (%)	N. samples positive *for *L. species* /N. samples positive for L. spp (%)	N. samples positive *for *mixed colture /N. samples positive for L. spp (%)
**Health - care**	Public Hospitals	705/2250 (31.3)	1264/2250 (56.2)	86/2250 (3.8)	195/2250 (8.7)
	
	Private Hospitals	54/176 (30.7)	66/176 (37.5)	37/176 (21.0)	19/176 (10.8)
	
	**Sub-total**	**759/2426 (31.3)**	**1330/2426 (54.8)**	**123/2426 (5.1)**	**214/2426 (8.8)**

**Community**	Apartments	36/81 (44.4)	7/81 (8.6)	20/81 (24.7)	18/81 (22.2)
	
	Hotels	588/1826 (32.2)	758/1826 (41.5)	79/1826 (4.3)	401/1826 (22.0)
	
	Offices	27/78 (34.6)	47/78 (60.3)	0/78 (0)	4/78 (5.1)
	
	Churches	1/3 (33.3)	1/3 (33.3)	1/3 (33.3)	0/3 (0)
	
	Gyms	20/46 (43.5)	16/46 (34.8)	10/46 (21.7)	0/46 (0)
	
	Swimming pools	1/1 (100)	0/1 (0)	0/1 (0)	0/1(0)
	
	Schools	0/3 (0)	2/3 (66.7)	1/3 (33.3)	0/3 (0)
	
	**Sub-total**	**673/2038 (33.0)**	**831/2038 (40.8)**	**111/2038 (5.4)**	**423/2038 (20.8)**

**Total**		**1432/4464 (32.1)**	**2161/4464 (48.4)**	**234/4464 (5.2)**	**637/4464 (14.3)**

* *L. longbeachae, L. bozemanii, L. dumoffii, L. gormanii, L. jordanis, L. micdadei, L. anisa*

**Figure 1:**
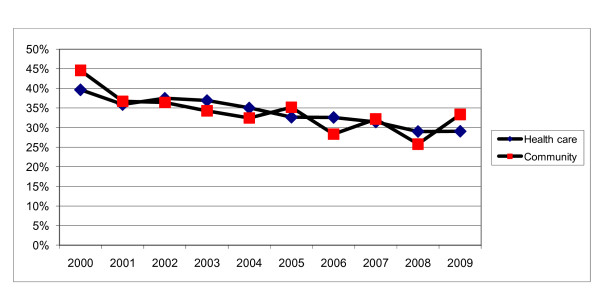
**Ten-year trend of percentage of positive samples collected from the community and health care facilities**.

Figure 1 shows the 10-year environmental surveillance trend of positive samples both for community and health care facilities.

**Figure 2:**
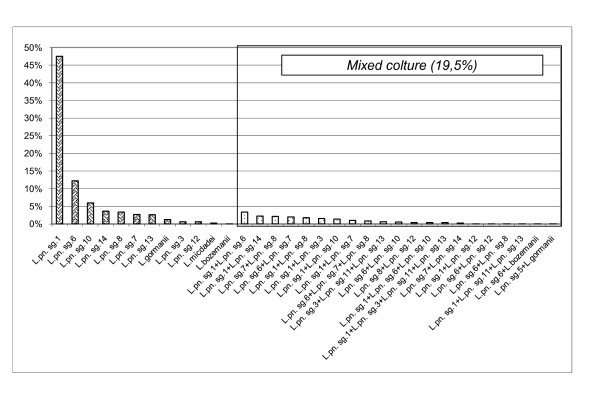
**Monovalent serotyping of *Legionella *spp. in health care facilities (years 2006-2009)**.

Health-care facilities were positive in 33.9% of samples and, of these, the percentages for the contamination levels <1,000, 1,000-10,000, > 10,000 cfu/L were respectively 33.1%, 53.4% and 13.5% (Table 3). Considering the results of the 10-year health-care facilities surveillance the most common species were *L. pn *sg 2-14 (54.8%), followed by *L. pn *sg 1 (31.3%), *L. species *(5.1%), mixed cultures (8.8%) (Table 4). While for monovalent antisera identification, carried out since 2006: *L. pn *sg1 (47.5%); *L. pn *sg 6 (12.2%); *L. pn *sg10 (5.9%) *L. pn *sg14 (3.6%) *L. pn *sg 8 (3.3%) *L. pn *sg7 (2.7%) *L. pn *sg13 (2.6%) *L. gormanii *(1.2%) *L. pn *sg3 (0.6%) *L. pn *sg12 (0.6%) *L. micdadei *(0.2%) *L. bozemanii *(0.1%); mixed cultures (19.5%) (Figure 2).

Buildings within the community were positive in 33.2% of samples and, of these, the percentages for the previously indicated contamination levels were respectively 33.5%, 43.3% and 23.2% (Table 3). The most common species were *L. pn *sg 2-14 (40.8%), *L. pn *sg 1 (33%), *L. species*. (5.4%), mixed culture (20.8%) (Table 4).

### Risk analysis

In total 67 health-care facilities (35 public hospital and 32 private hospital) and 283 community buildings (144 hotels, 72 gyms, 33 apartments, 21 schools, 10 offices, 3 swimming pools) underwent to a risk analysis: 191 (54.6%) buildings showed a G score, 159 (45.4%) an M or B score. The association between the results of risk analysis (G *vs *M or B score) and the results of the environmental surveillance (positive *vs *negative facilities) showed that the risk for a facility to test positive for *Legionella *spp. was higher in case of M or B score at risk analysis: RR 1.62; 95% CI 1.30-2.02; p < 0.001 (Table 5).

**Table 5:** Association between inspection scores and facilities positive for *Legionella *spp. (years 2005-2009)

G grade result		M or B grade result		RR of positive result by grade result (95% CI)	P value
		
Positive facilities (Total)	Rate	Positive facilities (Total)	Rate		
72 (191)	37.7	97 (159)	61	1.62 (1.30-2.02)	< 0.001

### Clinical Surveillance

In the period of the study, 97 cases of legionellosis were reported to the OER: 88 of community origin and 9 nosocomial: patient average age was 59.2 years (range 29-90), and 75.3% were males. Their occupations were: retired (38.6%), office workers (14.6%), factory workers (9.6%), professionals (9.6%), storekeepers (12%), artisans (3.6%), other (12%). Clinical symptoms are reported in table 6. Radiological evidence of pneumonia and pleural effusion was reported in 68% and 14.4% of cases respectively.

**Table 6:** Symptoms reported by patients affected by legionellosis


**Symptoms**	**N. of patients showing the symptoms/N. of total patients (%)**

Fever	91/97 (93.8%)

Couth	68/97 (70.1%)

Dyspnea	57/97 (58.8%)

Shivering	55/97 (56.7%)

Expectorating	41/97 (42.3%)

Headache	32/97 (33%)

Chest pain	31/97 (32%)

Nausea/vomiting	20/97 (20.6%)

Diarrhea	13/97 (13.4%)

Hemoptysis	11/97 (11.3%)

stomachache	11/97 (11.3%)

Other	29/97 (29.9%)

The laboratory diagnostic methods used were: urinary Ag (54.3%), single antibody titer (19.8%), only seroconversion (11.1%), antigenuria associated with single antibody titer (6.2%), antigenuria associated with seroconversion (4.9%), other associations of diagnostic methods (3.7%). The patients had one or more prior pathologies: cardiovascular (29.9%), diabetes (17.5%), respiratory (16.5%), neoplastic (8.3%), other endocrine (7.2%), genitourinary (6.2%), digestive (5.1%), other (18.6%).

There were no significant differences between regional community and regional nosocomial patients for age, therapy (fluoroquinolones were the most commonly prescribed antibiotics) and diagnostic method (urinary antigen and specific antibodies detection were the most common tests), while there was a significant difference for gender: males were 33.3% of nosocomial cases but 79.5% of cases of community origin (p < 0.05).

**Figure 3:**
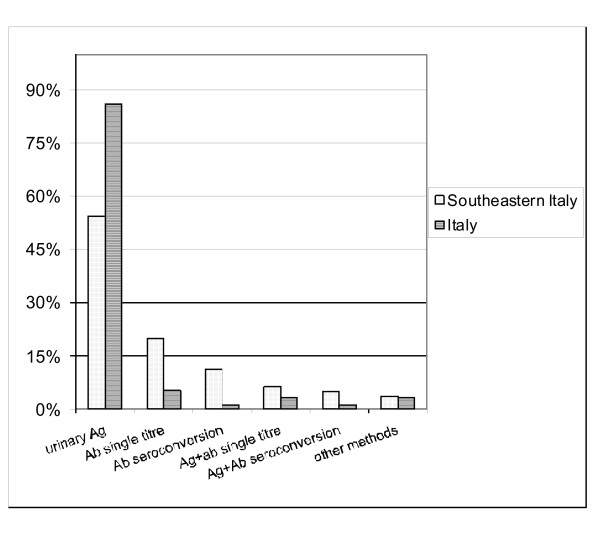
**Diagnostic methods (%) of legionellosis in Southeastern Italy and in Italy**.

Comparing regional and national data, Figure 3 shows the frequency of diagnostic methods used. The reason for hospital admission was significantly different between regional and national nosocomial cases: in Southeastern Italy there were less transplants and more infectious diseases (p < 0.05).

In one nosocomial case caused by *L. pn *sg 5, genotype comparison (through Pulsed Field Gel Electrophoresis) between the strain isolated from the patient and those isolated from the two different wards where the patient had been, both contaminated by *L. pn *sg 5, allowed the source to be identified [17].

## Discussion

The present surveillance program has shown a widespread environmental contamination of water systems by *Legionella *spp. The microorganism was found in 33.9% and 33.2% of the water samples coming respectively from health-care and community buildings. The 10-year environmental surveillance trend shows that, for both health-care and community buildings, the percentage of positive samples is decreasing: around 10% less from 2000 to 2009. But there is still room for improvement in the level of environmental contamination. In light of these data, the regional government is planning the production of its own Guidelines to take into account the specific characteristics of the region.

There was at least one sample positive for *Legionella *spp. in 66.9% of the hotels, which is in line with the findings in Italy and Europe with a contamination range from 63.6% to 75% [1,18,19]. Lower positive percentages have been reported by other authors: 20.8% in a Greek multicentric study, but the detection limit of the microbiological examination procedure was 500 ufc/l [20]. *L. pn*. sg. 2-14, was the principal isolate from our hotels but, our experience evidenced a high percentage (22%) of mixed *Legionella *spp. cultures. This finding confirms what was reported in a large Italian multicentric study [1], where, among 30 positive hotels, 6 showed mixed cultures. The percentage of hotels sampled in this study with a *Legionella *count > 10.000 ufc/L was higher (24.8%) than that found in the European literature (11.8-17.4% in Italy, 7.8% in Turkey and 4.2% in Greece) [1,18-20].

In a previous study of water samples from apartments in Italy, *Legionella *contamination ranged from 22.6% to 30.5% of the samples [2,18]; while the present study had a higher percentage of positive samples (42.9%).

In health care facilities, private hospitals were less contaminated than public ones: 60.7% vs 93.2%. The literature shows positive results in 100% hospitals in Italy [18], 62.5% in Taiwan [21] and 61.5% in Greece [22].

*L. pn *sg.1 was the most frequently isolate with 80% of the positives in hospitals in Taiwan and 72.5% of the isolated strains in Greece. In Italy, a study in one hospital in Rome identified *L. pn *sg 1 in 50% of the positive samples [23] while in another study in Bologna *L. pn *sg 1 was not identified [18]. In our study, the most frequently isolated species were *L. pn *sg 2-14 (54.8% of positive samples). However, through monovalent serotyping (Figure 1), we have found that in hospitals the most prevalent serogroup was *L. pn *sg 1 followed by *L. pn *sg 6 which is the second most virulent serogroup [24] and the second most frequently isolated strain in hospitals [21,23]. We can say that the pool serogroups *L. pn *sg 2-14 is, in reality, too large to be used to obtain accurate epidemiological data. It is desirable that monovalent serotyping of the isolates becomes a standard.

Though the literature states that *L. pn *sg 1 is the most common isolate in humans, more and more cases are being attributed to other species and serogroups. In particular a large European study on 1,335 strains isolated from human cases has shown that 33.9% of hospital acquired infection were caused by *Legionella *non-pneumophila 1 [25]; for this reason environmental contamination by L. non-pneumophila 1 should not be under-evaluated.

The *Legionella *spp. count was > 10,000 cfu/L in 13.5% of our positive hospital samples. Various guidelines, including those in Italy, recommend disinfection at this contamination threshold, even in absence of cases of disease [6]. However, it is important to underline that the simple measurement of colony forming units does not give the real estimate of the infection risk. In fact, the concentration of *Legionella *spp. from the hospital water system is not necessarily constant over time [26]. On the contrary, a risk assessment evaluation could be useful to predict *Legionella *spp. contamination in water systems. In fact, in the present study even if the standardized questionnaire used for the risk assessment is a short report including just 18 items, it was useful for the rapid evaluation of the principal environmental risk factors and to show that there is a clear correlation between the presence of a medium or high level of risk (M/B grading) and a positive result in environmental analysis for *Legionella *spp. Other Authors have developed a more precise standardized score inspection system, demonstrating a better reliability in predicting *Legionella *spp. proliferation in the water systems and in preventing Legionnaire disease [9]. In light of these data, in Southeastern Italy, the OER staff is working on an improved of risk assessment standardized report.

From a clinical point of view, our study underlines that the actual incidence of the disease has increased, especially if we compare the data in Southeastern Italy for the years 2001-2009 (97 reported cases) and for the years 1997-2000 (7 reported cases) [4]. One of the reasons for disease underestimation is the general lack of an etiological diagnosis for pneumonia. The patient often undergoes treatment without the actual cause being identified. In fact a study has shown that, in Italy, only in 11.2% of pneumonia cases was the etiological diagnosis indicated on the patient's records [27]. This problem is common to other infectious diseases, however it is more serious in the case of legionellosis which is subject to environmental surveillance to avoid further cases of the disease.

Though the diagnosis of legionellosis shows some difficulties (often it is not a routine laboratory practice, urine antigen emission is not constant, the antibody response is slow, etc), constant surveillance is necessary for possible cases of legionellosis. Not only should it be suspected in all pneumonia cases, but all specific laboratory tests should be carried out to clarify the suspicion. It has, in fact, been shown that legionellosis cannot be excluded by a negative urine antigen or by a single low-titre serological test. In our study, some patients were positive to only one of the tests recommended by the Italian Guidelines.

The isolation and identification of the etiological agent is fundamental to reach the source of infection and to program the necessary disinfection measures, so limiting the spread of the disease in both patients and health staff [28,29].

Our results suggest that a multi-professional approach must be taken for the control and management of *Legionella *spp. in water systems, with risk assessments and integrated risk management programs, involving and training all those who have to be aware of the legionellosis problem.

## Conclusions

Risk analysis and microbiological surveillance should be more frequent to control the environmental spread of *Legionella *spp. Moreover, considering that the emission of *Legionella *from water systems is not necessarily constant over time [26], the disinfection should be carried out, even in presence of low levels of contamination. This is particular true for health care facilities where people are more susceptible to infection.

From an epidemiological point of view, the underestimation of the disease suggests that a better diagnostic scheme is necessary for possible cases of legionellosis and that all the specific laboratory tests must be carried out to enable a correct diagnosis.

## Competing interests

The authors declare that they have no competing interests.

## Authors' contributions

CN and MTM contributed equally to this work from 2000; FF, RI, GB, and TC contributed to the present research from 2005. All the Authors have read and approved the final manuscript.

## Pre-publication history

The pre-publication history for this paper can be accessed here:

http://www.biomedcentral.com/1471-2458/10/660/prepub
